# Applications of Artificial Intelligence in the Endoscopic Detection and Characterization of Early Esophageal Squamous Cell Carcinoma: A Scoping Review

**DOI:** 10.3390/cancers18142235

**Published:** 2026-07-12

**Authors:** Faure Rodríguez-Velásquez, Andrés Montoya-Durán, Nicole Bonilla, Jacobo Echeverri-Hoyos, Jaime A. Echeverri-Franco, Eduardo Tuta-Quintero

**Affiliations:** 1 Department of Gastroenterology, School of Medicine, Universidad de La Sabana, Chía 250001, Colombia; andres.montoya2@unisabana.edu.co; 2 Endogut Research Group, Bogotá 111121, Colombia; 3 Gastroenterology Service, Fundación Clínica Shaio, Bogotá 111121, Colombia; 4 School of Medicine, Universidad de La Sabana, Chía 250001, Colombia; nicolebosi@unisabana.edu.co; 5 School of Medicine, Institución Universitaria Visión de las Américas, Pereira 660000, Colombia; jacoboecheverri9@gmail.com; 6 Pulmonology, Clínica de Alta Tecnología Oncólogos del Occidente, Pereira 660000, Colombia; jaime.echeverrif@gmail.com; 7 Department of Epidemiology and Internal Medicine, School of Medicine, Universidad de La Sabana, Chía 250001, Colombia; eduardotuqu@unisabana.edu.co

**Keywords:** esophageal cancer, cancer, diagnostic, artificial intelligence, scoping review

## Abstract

Background: Esophageal cancer is a highly lethal malignancy, the prognosis of which depends largely on early diagnosis. Artificial intelligence (AI) has emerged as a promising tool to enhance endoscopic detection and characterization of early esophageal cancer. This scoping review aims to map and synthesize the available evidence regarding the diagnostic performance and clinical utility of artificial intelligence systems applied to upper gastrointestinal endoscopy for the detection and characterization of premalignant squamous lesions and early-stage esophageal squamous cell carcinoma (ESCC). Methods: A scoping review was conducted according to Arksey and O’Malley, Levac, Joanna Briggs Institute, and PRISMA-ScR recommendations. The review question focused on patients with premalignant lesions or early ESCC, artificial intelligence-based diagnostic systems, and upper gastrointestinal endoscopy. Searches were performed in PubMed, Scopus, and Embase. Original studies reporting sensitivity, specificity, accuracy, AUC, or F1-score were included. Results: A total of 30 publications were included, consisting mainly of retrospective observational and diagnostic test studies (26/30; 86.7%), followed by randomized clinical trials (3/30; 10.0%) and a multicenter validation study (1/30; 3.3%). The studies were predominantly from China (18/30; 60%), followed by Japan (8/30; 26.7%), Taiwan (3/30; 10%), and the United Kingdom + Taiwan (1/30; 3%). Automatic lesion detection was predominant (21/30; 70.0%), followed by diagnostic classification (11/30; 36.7%), while segmentation (3/30; 10.0%), histological prediction (2/30; 6.7%), estimation of invasion depth (3/30; 10.0%), and lesion delineation (1/30; 3.3%) were evaluated less frequently, and in some cases combined within the same model. The most used endoscopic imaging modalities were narrow-band imaging (23/30; 76.7%) and white light endoscopy (20/30; 66.7%), followed by magnifying endoscopy with narrow-band imaging (5/30; 16.7%), blue light imaging (2/30; 6.7%), and hyperspectral imaging (1/30; 3.3%). Conclusions: Available studies suggest that AI has the potential to achieve high diagnostic performance under controlled conditions. However, the current evidence is derived predominantly from single-center retrospective studies using selected high-quality static images, with limited external, prospective, and real-world validation.

## Simple Summary

Early esophageal squamous cell carcinoma and its precancerous lesions can be difficult to recognize during endoscopy, especially in their earliest stages, when treatment is more effective and less invasive. In recent years, artificial intelligence has been developed to support endoscopists by helping detect suspicious lesions, classify their features, estimate invasion depth, and improve real-time decision-making. However, the available evidence is scattered across different technologies, imaging methods, and diagnostic tasks. This scoping review maps and summarizes current studies on artificial intelligence for the endoscopic diagnosis of early esophageal squamous cell carcinoma and related squamous precursor lesions. Overall, the evidence suggests that artificial intelligence can achieve high diagnostic performance and may improve lesion detection, support less experienced endoscopists, and reduce missed lesions. These findings help clarify the current state of the field, identify research gaps, and support the design of future validation and implementation studies.

## 1. Introduction

Esophageal cancer is currently one of the leading global cancer problems, ranking among the most common malignant neoplasms and accounting for a significant proportion of cancer deaths worldwide [1,2]. Epidemiological projections indicate that by 2040, there could be nearly 957,000 new diagnoses and approximately 880,000 deaths associated with this disease [2]. Esophageal Squamous Cell Carcinoma (ESCC) is the most common type, accounting for approximately 85% of esophageal cancer cases [2,3]. Its prognosis is closely related to the timing of diagnosis, as detection in early stages significantly improves treatment outcomes and survival [2,3,4]. The high mortality associated with this neoplasm, compared to other malignant tumors, underscores the need to strengthen early detection strategies [1,2,3,4].

Upper gastrointestinal endoscopy is the reference method for the detection and screening of precancerous lesions and early-stage neoplasms in the esophagus [5,6]. Although it is the current diagnostic standard, its performance may be influenced by observer-dependent factors, particularly in the identification of early lesions with subtle mucosal changes or inconspicuous vascular patterns [7,8,9]. Early diagnosis of esophageal cancer remains a challenge because many lesions present with subtle macroscopic findings on endoscopy, requiring a high level of expertise for their identification and targeted biopsy [8,9]. Currently, artificial intelligence (AI) and computer-aided diagnostic systems are emerging as promising tools to optimize this process by contributing to lesion detection, the classification of endoscopic patterns, margin delineation, and histological differentiation [10,11,12,13]. A previous systematic review and meta-analysis by Zhang et al. [14], which included 16 studies evaluating AI-assisted endoscopic detection of esophageal neoplasms, reported a pooled sensitivity of 94% and a pooled specificity of 85%, with AI demonstrating higher sensitivity than endoscopists (94% vs. 82%). However, the authors also highlighted that most included studies were retrospective and emphasized the need for prospective clinical validation before widespread implementation.

Despite the recent growth in evidence regarding AI applied to esophageal endoscopy, the available studies exhibit significant heterogeneity in terms of methodological designs, model architectures, endoscopic imaging modalities, and diagnostic performance metrics [13,14]. Most published studies have focused on superficial ESCC and related premalignant squamous lesions, whereas evidence regarding Barrett’s-associated neoplasia and esophageal adenocarcinoma remains comparatively limited. Differences also persist in the evaluation of specific tasks such as detection, classification, segmentation, and estimation of invasion depth [4,13,14]. In this context, the aim of this scoping review was to synthesize and compare the available evidence on the diagnostic performance of AI systems applied to the endoscopic detection and characterization of early ESCC, identifying their main clinical applications, diagnostic outcomes, and current methodological limitations.

## 2. Methods

### 2.1. Design of the Scoping Review

This scoping review was conducted in accordance with the methodological recommendations proposed by Arksey and O’Malley [15] and expanded upon by Levac [16], as well as the methodological guidelines of the Joanna Briggs Institute [17] for scoping reviews. The report was prepared in accordance with the PRISMA Extension for Scoping Reviews (Preferred Reporting Items for Systematic Reviews and Meta-Analyses extension for Scoping Reviews) [18] (Supplementary File S1), with the aim of mapping and synthesizing the available evidence on the use of AI applied to the endoscopic diagnosis of early ESCC and its precursor lesions.

The review protocol was prospectively registered in the Open Science Framework (OSF) to promote methodological transparency and reproducibility. The registered protocol is available at: Tuta-Quintero E et al. Applications of Artificial Intelligence in the Endoscopic Detection and Characterization of Early Esophageal Squamous Cell Carcinoma: A Scoping Review. Open Science Framework. Available online: https://osf.io/3rmsk/overview (accessed on 28 June 2026). 

### 2.2. Formulation of the Review Question

The review question was structured using the PCC (Population, Concept, and Context) approach [19]. The population consisted of patients with premalignant esophageal lesions, dysplasia, or early ESCC; the concept corresponded to artificial intelligence systems based on deep learning, machine learning, or computer-aided diagnosis; and the context was limited to the use of images or videos obtained via upper gastrointestinal endoscopy. Based on this, the guiding question was: What is the diagnostic performance of artificial intelligence systems applied to endoscopic images for the detection, classification, segmentation, or estimation of invasion in early esophageal cancer?

### 2.3. Literature Search Strategy

The literature search strategy was designed to identify studies published in international biomedical databases, including PubMed, Scopus, and Embase [15,16,17,18,19]. Controlled terms and keywords related to artificial intelligence, deep learning, convolutional neural networks, esophageal cancer, early esophageal neoplasia, and upper gastrointestinal endoscopy were used. Search combinations included Boolean operators to maximize sensitivity and specificity in retrieving potentially relevant studies (Supplementary File S2).

### 2.4. Eligibility Criteria

We included original studies evaluating artificial intelligence systems applied to the endoscopic diagnosis of premalignant squamous lesions and early ESCC, provided they reported diagnostic performance metrics such as sensitivity, specificity, diagnostic accuracy, area under the ROC curve, or F1-score. Observational studies, diagnostic test studies, multicenter validation studies, and randomized clinical trials were considered eligible. Studies using endoscopic imaging modalities, including white light endoscopy (WLE), narrow-band imaging (NBI), magnifying endoscopy with narrow-band imaging (ME-NBI), blue light imaging (BLI), and other advanced endoscopic techniques applied to esophageal squamous lesions were included. Narrative reviews, editorials, studies without quantifiable diagnostic outcomes, and publications focused exclusively on technical development without clinical validation were excluded. Studies primarily focused on Barrett’s-associated neoplasia or esophageal adenocarcinoma were not the focus of this review and were therefore not specifically targeted in the search strategy. When such studies were identified, they were included only if they reported data specific to early ESCC or premalignant squamous lesions.

### 2.5. Definition of the Main Diagnostic Tasks Evaluated by AI

The tasks performed by the artificial intelligence systems included in this review were classified according to their primary diagnostic function. Detection refers to the automatic identification of suspicious lesions in endoscopic images or videos; classification to the assignment of a diagnostic category based on endoscopic or histological characteristics; segmentation to the pixel-level identification of the lesion within the image; and lesion delineation to the identification of lesion boundaries or margins to define lesion extent. Likewise, histological prediction was defined as the estimation of the histological type or degree of tumor differentiation based on visual patterns, while invasion depth estimation referred to the automated assessment of tumor infiltration into the different layers of the esophageal wall.

### 2.6. Study Selection

Study selection was conducted in two sequential stages. In the first stage, titles and abstracts were reviewed to identify potentially eligible publications, which were then imported into the Rayyan platform [20], where two independent reviewers assessed the titles and abstracts using previously standardized eligibility criteria. Subsequently, a full-text review of the selected articles was conducted to confirm their final inclusion. Eligibility was determined based on the previously established criteria, with discrepancies resolved through methodological consensus.

### 2.7. Data Extraction

A previously designed standardized matrix was used for data extraction [21]. For each study, information was recorded regarding the author, year of publication, country of origin, study type, type of esophageal lesion or cancer evaluated, endoscopic imaging modality used, type of task performed by the artificial intelligence system, sample size, comparison with endoscopists, primary diagnostic performance metrics, relevant clinical findings, and methodological limitations reported by the authors.

### 2.8. Synthesis and Analysis of Evidence

The results were synthesized using a descriptive narrative approach, consistent with the objectives of a scoping review to map and characterize the breadth and nature of the available evidence rather than to quantitatively synthesize intervention effects. Accordingly, and in keeping with established scoping review methodology, no formal critical appraisal of methodological quality or risk of bias, nor a meta-analysis, was performed [15,16,17,18]. To improve the presentation of the findings, the results were organized into two tables according to the primary AI application: (1) lesion detection and (2) lesion classification and characterization. Some studies evaluated more than one AI task and are therefore presented in both tables according to their reported applications. Overall, the included studies encompassed automatic lesion detection, histological classification, lesion segmentation, lesion delineation, prediction of invasion depth, and real-time assessment during endoscopic procedures, reflecting the heterogeneity in study designs, AI model architecture, imaging modalities, sample sizes, and reported outcomes.

## 3. Results

**Figure 1:**
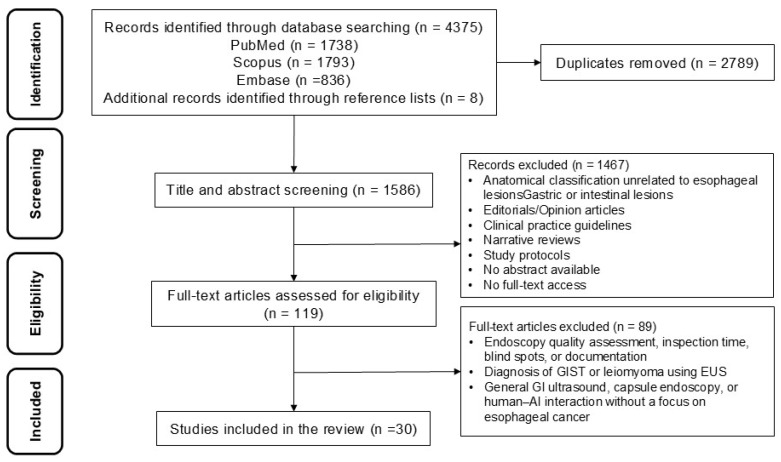
PRISMA Flowchart.

A total of 30 publications were included (Figure 1), consisting mainly of retrospective observational and diagnostic test studies (26/30; 86.7%), followed by randomized clinical trials (3/30; 10.0%) and a multicenter validation study (1/30; 3.3%) (Table 1 and Table 2) [22,23,24,25,26,27,28,29,30,31,32,33,34,35,36,37,38,39,40,41,42,43,44,45,46,47,48,49,50,51]. The studies were predominantly from China (18/30; 60%), followed by Japan (8/30; 26.7%), Taiwan (3/30; 10%), and the United Kingdom + Taiwan (1/30; 3%). Most studies focused on early ESCC and premalignant squamous lesions, whereas only a limited number included esophageal adenocarcinoma.

Regarding the type of AI task, automatic lesion detection was predominant (21/30; 70.0%), followed by diagnostic classification (11/30; 36.7%), while segmentation (3/30; 10.0%), histological prediction (2/30; 6.7%), estimation of invasion depth (3/30; 10.0%), and lesion delineation (1/30; 3.3%) were evaluated less frequently, and in some cases combined within the same model. The most used endoscopic imaging modalities were NBI (23/30; 76.7%) and WLE (20/30; 66.7%), followed by ME-NBI (5/30; 16.7%), BLI (2/30; 6.7%), and hyperspectral imaging (1/30; 3.3%).

The main limitations of the included studies were the predominance of retrospective single-center designs, the use of selected static high-quality images, limited real-time or prospective validation, small or imbalanced datasets, restricted lesion diversity, and limited generalizability due to dependence on specific imaging modalities and endoscopic platforms (Supplementary File S3).

Because the included studies addressed different AI tasks (e.g., detection, classification, segmentation, delineation, histological prediction, and invasion depth estimation), they reported heterogeneous performance metrics, including accuracy, sensitivity, specificity, area under the curve, F1-score, precision, recall, and lesion detection or miss rates (Table 1 and Table 2) [22,23,24,25,26,27,28,29,30,31,32,33,34,35,36,37,38,39,40,41,42,43,44,45,46,47,48,49,50,51]. Consequently, these metrics are presented descriptively and should not be interpreted as directly comparable across studies or AI applications.

Horie et al. [31] reported 98% accuracy and 98% sensitivity in the detection of squamous cell carcinoma and adenocarcinoma using a deep learning model trained on over 8000 images, confirming a high capacity for rapidly analyzing stored endoscopic images. Meng et al. [23], in a multicenter validation study involving more than 6000 images, reported an area under the curve of 0.982, an accuracy of 92.9%, a sensitivity of 91.9%, and a specificity of 94.7%, further demonstrating improved performance among non-expert endoscopists. Comparable results were observed by Tang et al. [32], with an area under the curve of 0.954, a sensitivity of 97.9%, and a negative predictive value of 99.1%, outperforming the evaluated endoscopists. Cai et al. [50] reported a sensitivity of 97.8% and an accuracy of 91.4% using WLE exclusively, showing that even in conventional modalities, artificial intelligence can detect previously unnoticed lesions.

Feng et al. [25] analyzed 9686 images and reported an internal sensitivity of 96.64% and an external sensitivity of 90.17%, with areas under the curve of 0.930 and 0.974, respectively, confirming diagnostic stability in external validation. Chou et al. [30] also reported an accuracy of 96.32%, with an F1-score of 96.04%, using a hybrid deep learning model, reinforcing the consistency of high performance in static images.

Wang J et al. [22] evaluated ME-BLI and ME-NBI images for the detection of intrapapillary capillary loops, achieving a recall of 79.25%, a precision of 75.54%, and an F1-score of 0.764, with adequate diagnostic generalization. Uema et al. [28] developed a system designed to classify vascular grades B1–B3 and invasion depth, achieving accuracies of 84.2% and 86.3% for invasion and outperforming human evaluators, while also drastically reducing diagnostic time.

Wang YK et al. [26] reported an overall accuracy of 92% in classifying histological grades, with better performance for NBI (95%) compared to WLE (89%). Tajiri et al. [39], using a Big Transfer architecture, achieved an accuracy of 80.9% and a sensitivity of 85.5%, outperforming endoscopists without assistance. Zhao et al. [24] reported 91% accuracy with Inception V3, comparable to expert physicians, but with significantly faster diagnostic speed (0.02 s vs. 5.65 s).

Yuan et al. [34] demonstrated accuracies between 87% and 89% for lesion extent under NBI, including prospective clinical validation with detection rates above 91%. Tang et al. [49] developed multitasking models capable of simultaneously classifying and segmenting esophageal lesions, with an accuracy of 93.43%, outperforming endoscopists.

Yuan et al. [44] demonstrated that AI-assisted analysis improved the classification of IPCL subtypes from 78.2% to 84.7% and increased the accuracy of invasion estimates from 67.9% to 74.4%. The sensitivity of AI was higher than that of junior operators and comparable to that of experts. Ohmori et al. [37] reported 100% sensitivity in non-magnified NBI compared to 92% in experts. Yuan et al. [51] observed higher sensitivity of AI compared to experts (90.8% vs. 82.5%). Gao et al. [42] demonstrated increased accuracy and reduced reading time for both junior and senior physicians when algorithmic assistance was used.

Yuan et al. [35] showed that the rate of missed lesions decreased from 6.7% to 1.7% with artificial intelligence assistance. Li et al. [36], using the ENDOANGEL system, demonstrated a detection rate of high-risk esophageal lesions of 1.8% compared to 0.9% in the control group, with an overall accuracy of 98.2%. Similarly, Li B et al. [40] reported a significant increase in the detection of esophageal neoplasms (3.12% vs. 1.59%) in a prospective randomized trial.

## 4. Discussion

Our review examined the available evidence on the diagnostic performance of AI systems applied to the endoscopic detection and characterization of early ESCC and premalignant squamous lesions. The evidence showed a predominance of retrospective observational and diagnostic test studies focused on the development and validation of AI systems for early ESCC diagnosis [22,23,24,25,26,27,28,29,30,31,32,33,34,37,38,39,40,41,42,43,44,45,46,47,48,49,50,51]. Only a limited number of studies corresponded to randomized clinical trials or multicenter validations [23,35,36,40]. Most studies originated from Asian countries, particularly China and Japan, reflecting the epidemiological predominance of ESCC in these regions [22,23,24,25,27,28,29,30,32,33,34,35,36,37,38,39,40,41,42,43,44,45,46,47,49,50,51].

The most-used imaging modalities were WLE, NBI, ME-NBI, BLI, and hyperspectral imaging systems [22,23,24,25,26,27,28,29,31,33,37,39,42,44,46,47,51]. The most frequently evaluated diagnostic task was automatic lesion detection, followed by diagnostic classification, segmentation, histological prediction, and estimation of invasion depth [22,23,24,25,26,27,28,29,30,32,33,34,35,36,37,38,39,40,41,42,43,44,45,46,47,48,49,51]. In general, deep convolutional neural network models demonstrated high diagnostic performance in static images and selected datasets [23,24,25,27,28,29,30,32,33,37,43,45,47,49,50].

Deep learning-based models achieved high diagnostic metrics in tasks involving the detection, classification, segmentation, and delineation of early ESCC lesions. Several studies reported area under the curve values exceeding 0.90 [23,24,25,28,32,33,50,51], suggesting that AI systems can identify endoscopic patterns associated with superficial squamous neoplasia with diagnostic performance comparable to that of expert endoscopists in some studies, while in others they outperformed less experienced endoscopists or were compared with endoscopists whose level of expertise was not explicitly reported [23,24,32,37,39,45,49,50,51]. However, most of these results were obtained under retrospective and highly controlled conditions, frequently using high-quality static images from specialized centers and excluding common real-world factors such as motion artifacts, secretions, variable illumination, or differences in endoscopic technique [23,24,25,28,30,32,37,43,45,47,49,50]. Consequently, the performance reported in experimental settings may overestimate the true effectiveness of these systems in routine clinical practice [23,28,30,37,41,43,45].

Although many studies reported high diagnostic performance, direct numerical comparisons across AI systems should be interpreted with caution. The included studies evaluated different clinical tasks using different performance metrics [22,23,24,25,26,27,28,29,30,32,33,34,35,36,39,44,45,48,49,50,51]. Furthermore, variations in study design, patient populations, imaging modalities, reference standards, and validation strategies [22,23,24,25,26,27,28,29,30,31,32,33,34,35,36,37,38,39,40,41,42,43,44,45,46,47,48,49,50,51] limit the comparability of the reported results. Therefore, the findings of this scoping review should be interpreted as a descriptive mapping of the current evidence rather than as a comparative assessment of AI performance across studies.

This discrepancy becomes more evident in studies incorporating video analysis or real-time validation, where technical limitations persist despite promising results [27,33,46,51]. Shiroma et al. demonstrated that performance declined during high-speed endoscopic videos, particularly with NBI modalities [27]. Similarly, Waki et al. reported high sensitivity but low specificity in video-based clinical validation, reflecting an increase in false-positive detections during continuous image analysis [46]. These findings indicate that the transition from controlled image-based algorithms to robust real-time clinical support systems remains a major challenge.

Most of the available evidence focuses on superficial ESCC and premalignant squamous lesions, whereas studies evaluating Barrett’s-associated neoplasia or esophageal adenocarcinoma remain scarce [31]. This histological imbalance reflects both the epidemiology of esophageal cancer in Asian countries and the current direction of AI research in esophageal endoscopy. Consequently, most AI systems have been trained using squamous epithelial patterns and may not be generalizable to Barrett’s esophagus-associated lesions or early adenocarcinoma.

Most AI systems were developed using high-resolution endoscopic platforms combined with advanced imaging modalities such as WLE, NBI, ME-NBI, and hyperspectral imaging [22,26,29,33,37,39,44,46,47,51]. This technological dependence may limit broader implementation in healthcare systems with restricted access to advanced endoscopic equipment [26,29,37,46]. Nevertheless, several studies demonstrated that AI assistance can improve diagnostic sensitivity, increase lesion detection rates, reduce reading time, and particularly support junior or less experienced endoscopists, whereas studies involving expert endoscopists generally reported comparable diagnostic performance or improvements in efficiency rather than clear superiority [35,36,38,42,44,50,51]. AI-assisted systems may also contribute to reducing interobserver variability and improving diagnostic standardization, particularly for subtle superficial lesions.

Randomized clinical trials have begun to provide evidence beyond purely algorithmic performance metrics. Yuan et al. demonstrated a significant reduction in missed lesions with AI assistance [35], while Li et al. reported increased detection rates of high-risk esophageal lesions using the ENDOANGEL system [36]. Similarly, Li B et al. showed significantly higher neoplasm detection rates with AI-assisted endoscopy compared with conventional examination alone [40]. These findings suggest potential clinical utility; however, current evidence remains insufficient to determine whether AI-assisted diagnosis ultimately improves long-term outcomes such as earlier therapeutic intervention, reduced tumor progression, or survival.

In tasks involving vascular classification, histological prediction, and invasion depth estimation, AI systems also demonstrated promising performance. Studies by Uema et al., Yuan et al., and Everson et al. showed high accuracy in classifying intrapapillary capillary loop patterns and predicting invasion depth [28,44,48]. These applications are clinically relevant because they may directly influence therapeutic decisions, including endoscopic resection strategies. However, current evidence remains insufficient to replace expert endoscopic assessment in these complex diagnostic tasks, and the level of endoscopist expertise was not consistently reported across all comparative studies, limiting direct comparisons.

Deep learning algorithms consistently improved lesion detection and diagnostic performance across multiple studies [23,24,25,32,35,36,40,50,51]. Recent reviews suggest that the field is progressively evolving toward multimodal machine learning approaches integrating imaging, genomic, transcriptomic, proteomic, and clinical data to improve diagnosis, prognostic stratification, therapeutic target identification, and personalized treatment selection [52]. However, despite these advances, most available evidence is based on retrospective, single-center datasets with limited external validation, highlighting persistent concerns regarding model generalizability, data heterogeneity, and clinical interpretability. These observations are consistent with our review, which found that prospective randomized studies and real-world implementation studies evaluating AI-assisted endoscopy in ESCC remain scarce [23,25,27,30,35,36,37,40,41,46].

### Limitations

Consistent with the objectives and methodology of a scoping review, we did not perform a formal critical appraisal of methodological quality or risk of bias of the included studies [17,18]. Consequently, the validity of individual studies and the certainty of the available evidence could not be formally assessed, and the findings should be interpreted as a descriptive mapping of the current literature rather than a quantitative evaluation of comparative effectiveness [15,16]. In addition, the marked heterogeneity in study designs, AI models, imaging modalities, populations, and reported outcomes precluded quantitative synthesis. Therefore, although several studies reported diagnostic performance comparable to that of endoscopists under specific conditions, these findings should not be interpreted as definitive evidence of clinical superiority. Dedicated systematic reviews with formal risk-of-bias assessment and meta-analysis of homogeneous prospective studies will be necessary to establish the comparative effectiveness and clinical utility of AI-assisted endoscopic diagnosis.

Importantly, these methodological limitations were not confined to isolated reports but were consistently observed across the available literature (Supplementary File S3). Recurrent issues highlight that these challenges are pervasive in the current evidence base and should be considered when interpreting the reported diagnostic performance of AI systems.

## 5. Conclusions

Available studies suggest that AI has the potential to achieve high diagnostic performance under controlled conditions. However, the current evidence is derived predominantly from single-center retrospective studies using selected high-quality static images, with limited external, prospective, and real-world validation. Importantly, only one randomized controlled trial has evaluated an AI algorithm for ESCC detection. These findings highlight the rapid evolution of the field while underscoring the need for well-designed multicenter prospective studies and randomized trials to establish the clinical utility, generalizability, and implementation of AI-assisted endoscopic diagnosis in routine practice.

## Figures and Tables

**Table 1:** Studies evaluating AI for lesion detection in early ESCC and premalignant squamous lesions.

Author	Country	Lesion Type	Imaging Modality	AI Task	Study Design	Main AI Performance
Wang J et al. [22]	China	Early ESCC/IPCLs	ME-NBI, ME-BLI	Detection	Observational diagnostic study	F1-score 0.764; Mean Average Precision 74.95%
Meng QQ et al. [23]	China	ESCC/Dysplasia	WLE, NBI	Detection/Segmentation	Multicenter validation	AUC 0.982; Acc 92.9%; Sen 91.9%; Spe 94.7%
Feng Y et al. [25]	China	Superficial ESCC	WLE	Detection	Observational study	Internal: AUC 0.930; Acc 91.75%; Sen 96.64%; Spe 95.35%. External: AUC 0.974; Acc 88.38%; Sen 90.17%; Spe 94.34%
Wang YK et al. [26]	Taiwan	ESCC/Dysplasia	WLE, NBI	Detection/Classification	Observational study	Detection: Acc 90.9%; Sen 96.2%; Spe 70.4%
Shiroma S et al. [27]	Japan	ESCC	WLE, NBI	Detection	Observational study	Slow-motion: Sen 100%; High-speed: overall Sen 85%
Chou CK et al. [30]	Taiwan	ESCC/Dysplasia	WLE, NBI	Detection	Observational study	Acc 96.3%; Recall 95.7%; F1-score 96.04%
Horie Y et al. [31]	Japan	ESCC/Adenocarcinoma	WLE, NBI	Detection	Observational study	Acc 98%; Sen 98%; PPV 40%; NPV 95%
Tang D et al. [32]	China	Early ESCC	WLE	Detection	Observational study	AUC 0.954; Sen 97.9%; Spe 88.6%
Guo L et al. [33]	China	Precancerous lesions/Early ESCC	NBI	Detection	Observational study	AUC 0.989; Sen 98.04%; Spe 95.03%
Yuan XL et al. [34]	China	Precancerous lesions/ESCC	NBI	Detection/Delineation	Observational study	Detection Acc 91.4%; Delineation Acc 85.9%
Yuan XL et al. [35]	China	Superficial ESCC	WLE, NBI	Detection	Randomized clinical trial	Missed lesion rate: 1.7% vs. 6.7%
Li SW et al. [36]	China	Superficial ESCC	WLE, NBI, ME	Detection	Randomized clinical trial	Acc 98.2%; Sen 89.7%; Spe 98.5%
Ohmori M et al. [37]	Japan	Superficial ESCC	WLE, NBI	Detection/Classification	Observational study	Acc 77%; Sen 100%; Spe 63%
Aoyama N et al. [38]	Japan	Superficial ESCC	NBI	Detection	Observational study	Acc 77.2%; Sen 57.4%; Spe 87.0%
Li B et al. [40]	China	Precancerous lesions/ESCC	NBI	Detection	Prospective randomized trial	Detection rate 3.12%
Tani Y et al. [41]	Japan	ESCC	WLE, NBI	Detection	Observational study	Acc 80.6%; Sen 68.2%; Spe 83.4%
Waki K et al. [46]	Japan	ESCC	WLE, NBI, BLI	Detection	Observational study	Sen 85.7%; Spe 40%
Li B et al. [47]	China	Early ESCC	WLE, NBI	Detection	Observational study	CAD-NBI Acc 94.3%; CAD-WLI Acc 89.5%
Everson MA et al. [48]	United Kingdom/Taiwan	Early ESCC/IPCLs	ME-NBI	Detection/Histological prediction	Observational study	F1-score 94%; Sen 93.7%; Acc 91.7%
Cai et al. [50]	China	ESCC	WLE	Detection	Observational study	DNN-CAD Acc 91.4%; Expert 88.8%; Junior 77.2%
Yuan XL et al. [51]	China	ESCC	WLE, NBI, ME-NBI	Detection	Observational study	AI Sen 90.8% vs. Expert Sen 82.5% (*p* = 0.022)

Notes: ESCC, esophageal squamous cell carcinoma; IPCLs, intrapapillary capillary loops; WLE, white-light endoscopy; NBI, narrow-band imaging; ME, magnifying endoscopy; ME-NBI, magnifying endoscopy with narrow-band imaging; ME-BLI, magnifying blue-light imaging; BLI, blue-light imaging; AUC, area under the curve; Acc, accuracy; Sen, sensitivity; Spe, specificity; PPV, positive predictive value; NPV, negative predictive value; DNN-CAD, deep neural network computer-aided diagnosis.

**Table 2:** Studies evaluating AI for lesion classification and characterization in early ESCC and premalignant squamous lesions.

Author	Country	Lesion Type	Imaging Modality	AI Task	Study Design	Main AI Performance
Zhao Z et al. [24]	China	Early ESCC	NBI	Classification	Observational diagnostic study	IA-NBI: AUC 0.910; Acc 91%; Physician AUC 0.930
Wang YK et al. [26]	Taiwan	ESCC/Dysplasia	WLE, NBI	Classification	Observational study	Histological classification: Acc 92% (NBI 95%; WLE 89%)
Uema R et al. [28]	Japan	Superficial ESCC	NBI	Classification/Depth estimation	Observational study	Classification Acc 84.2%; Depth estimation Acc 86.3%
Wang YK et al. [29]	Taiwan	ESCC/Dysplasia	WLE, NBI, HSI	Classification	Observational study	WLE Acc 83%; NBI Acc 82%; HSI Acc 89–90%
Ohmori M et al. [37]	Japan	Superficial ESCC	WLE, NBI	Classification	Observational study	Acc 77%; Sen 100%; Spe 63%
Tajiri A et al. [39]	Japan	Superficial ESCC	NBI, ME	Classification	Observational study	Acc 80.9%; Sen 85.5%; Spe 75.0%
Gao X et al. [42]	China	Early esophageal cancer	WLE, NBI, ME-NBI	Classification	Observational study	Improved diagnostic accuracy with AI assistance for junior and senior endoscopists
Yang XX et al. [43]	China	ESCC	WLE, ME	Classification	Observational study	Non-ME WLE Acc 99.5%; ME Acc 88.1%
Yuan XL et al. [44]	China	Precancerous lesions/ESCC	ME-NBI	Classification/Depth estimation	Observational study	IPCL classification Acc 91.3% (internal); 89.8% (external)
Tang S et al. [45]	China	Esophageal lesions	WLE, NBI	Classification/Segmentation	Observational study	Acc 95.9%; Sen 95.39%; Spe 97.73%
Tang et al. [49]	China	Esophageal lesions	WLE, NBI	Classification/Segmentation	Observational study	Acc 93.4%; Sen 92.82%; Spe 96.20%

Notes: ESCC, esophageal squamous cell carcinoma; WLE, white-light endoscopy; NBI, narrow-band imaging; ME, magnifying endoscopy; ME-NBI, magnifying endoscopy with narrow-band imaging; HSI, hyperspectral imaging; IPCL, intrapapillary capillary loop; AUC, area under the curve; Acc, accuracy; Sen, sensitivity; Spe, specificity.

## Data Availability

The datasets used in this study can be found in the full-text articles that were included in the scoping review.

## Supplementary Materials

The following supporting information can be downloaded at https://www.mdpi.com/article/10.3390/cancers18142235/s1, Supplementary File S1: PRISMA Extension for Scoping Reviews (PRISMA-ScR) 2018 Checklist [18]; Supplementary File S2: Search Strategies (search updated 1 January 2026); Supplementary File S3: Main methodological limitations of included studies [22,23,24,25,26,27,28,29,30,31,32,33,34,35,36,37,38,39,40,41,42,43,44,45,46,47,48,49,50,51].
