# Supplementary material for: Applications of Artificial Intelligence in the Endoscopic Detection and Characterization of Early Esophageal Squamous Cell Carcinoma: A Scoping Review
Source: Cancers (Basel). 2026 Jul 12;18(14):2235. doi: 10.3390/cancers18142235 (PMC13406400; doi:10.3390/cancers18142235)
Supplement: Supplementary file 1 [file cancers-18-02235-s001.zip › cancers-4341417-supplementary.pdf]

Supplementary File S1. PRISMA Extension for Scoping Reviews (PRISMA-ScR) 2018 Checklist[1]

Section/topic	#	PRISMA-ScR Checklist item	Reported on page #
**TITLE**			
Title	1	Identify the report as a scoping review.	1
**ABSTRACT**			
Structured summary	2	Provide a structured summary including, as applicable: background; objectives; data sources; study eligibility criteria, participants, and interventions; study synthesis methods; results; limitations; conclusions and implications of key findings.	1
**INTRODUCTION**			
Rationale	3	Describe the rationale for the review in the context of what is already known. Explain why the review question(s)/objective(s) lend themselves to a scoping review approach.	2
Objectives	4	Provide an explicit statement of the question(s) and objective(s) being addressed with reference to their key elements (e.g., population or participants, concepts and context), or other relevant key elements used to conceptualize the review question(s) and/or objective(s)).	2
**METHODS**			
Protocol and registration	5	Indicate if a review protocol exists, if and where it can be accessed (e.g., Web address), and, if available, provide registration information including registration number.	3
Eligibility criteria	6	Specify the characteristics of the sources of evidence (e.g., years considered, language, publication status) used as criteria for eligibility, and provide a rationale.	3
Information sources	7	Describe all information sources (e.g., databases with dates of coverage, contact with study authors to identify additional sources) in the search and date last searched.	3
Search	8	Present full electronic search strategy for at least one database, including any limits used, such that it could be repeated.	Supplementary file
Selection of sources of evidence	9	State the process for selecting studies (i.e., screening, eligibility) included in the scoping review.	4
Data charting process	10	Describe the methods of charting data from the included sources of evidence (e.g. piloted forms; forms that have been tested by the team before their use, whether data charting was done independently, in duplicate) and any processes for obtaining and confirming data from investigators.	4
Data items	11	List and define all variables for which data were sought and any assumptions and simplifications made.	5
Critical appraisal of individual	12	**If done,** provide a rationale for conducting a critical appraisal of included sources of evidence; describe the methods used and how	Not applicable

Section/topic	#	PRISMA-ScR Checklist item	Reported on page #
sources of evidence		this information was used in any data synthesis (if appropriate).	
Summary measures	13	Not applicable for scoping reviews.	Not applicable
Synthesis of results	14	Describe the methods of handling and summarizing the data that were charted.	4
Risk of bias across studies	15	Not applicable for scoping reviews.	Not applicable
Additional analyses	16	Not applicable for scoping reviews.	Not applicable
**RESULTS**			
Selection of sources of evidence	17	Give numbers of studies screened, assessed for eligibility, and included in the review, with reasons for exclusions at each stage, ideally using a flow diagram.	Figure 1<br>5-8
Characteristics of sources of evidence	18	For each source of evidence, present characteristics for which data were charted and provide the citations.	Table 1 and table 2<br>5-8
Critical appraisal within sources of evidence	19	If done, present data on critical appraisal of included sources of evidence (see item 12).	Not applicable
Results of individual sources of evidence	20	For each included source of evidence, present the relevant data that were charted that relate to the review question(s) and objective(s).	Table 1 and table 2<br>5-8
Synthesis of results	21	Summarize and/or present the charting results as they relate to the review question(s) and objective(s).	Table 1 and<br>5-8
Risk of bias across studies	22	Not applicable for scoping reviews.	NA
Additional analysis	23	Not applicable for scoping reviews.	NA
**DISCUSSION**			
Summary of evidence	24	Summarize the main results (including an overview of concepts, themes, and types of evidence available), explain how they relate to the review question(s) and objectives, and consider the relevance to key groups	9-10
Limitations	25	Discuss the limitations of the scoping review process.	10
Conclusions	26	Provide a general interpretation of the results with respect to the review question(s) and objective(s), as well as potential implications and/or next steps.	11
**FUNDING**			

Section/topic	#	PRISMA-ScR Checklist item	Reported on page #
Funding	27	Describe sources of funding for the included sources of evidence, as well as sources of funding for the scoping review. Describe the role of the funders of the scoping review.	11

[18] Tricco AC, Lillie E, Zarin W, et al. PRISMA Extension for Scoping Reviews (PRISMA-ScR): Checklist and Explanation. *Ann Intern Med* 2018;169(7):467-73.

Supplementary File S2. Search Strategies (search updated January 1, 2026)

PubMed/MEDLINE
("artificial intelligence"[tiab] OR AI[tiab] OR "machine learning"[tiab] OR "deep learning"[tiab] OR "neural network*"[tiab] OR "convolutional neural network*"[tiab] OR CNN[tiab] OR "computer-aided"[tiab] OR "computer aided"[tiab] OR CADe[tiab] OR CADx[tiab] OR "clinical decision support"[tiab]) AND (endoscop*[tiab] OR "upper endoscopy"[tiab] OR gastroscop*[tiab] OR esophagogastroduodenoscop*[tiab] OR EGD[tiab] OR "capsule endoscop*"[tiab] OR "video capsule"[tiab] OR VCE[tiab] OR "endoscopic ultrasound"[tiab] OR EUS[tiab] OR "gastrointestinal bleeding"[tiab] OR "upper gastrointestinal bleeding"[tiab] OR GI bleed*[tiab]) AND (esophag*[tiab] OR stomach[tiab] OR gastric[tiab] OR duoden*[tiab] OR upper[tiab] OR small bowel[tiab] OR enter*[tiab] OR Barrett*[tiab] OR "early gastric cancer"[tiab] OR varice*[tiab] OR ulcer*[tiab] OR "subepithelial"[tiab] OR "gastrointestinal stromal"[tiab] OR GIST[tiab]) Filters: from database inception to January 1, 2026.
Scopus
TITLE-ABS-KEY ("artificial intelligence" OR "machine learning" OR "deep learning" OR "convolutional neural network*" OR "neural network*" OR "computer aided detection" OR "computer aided diagnosis") AND TITLE-ABS-KEY (endoscop* OR gastroscop* OR esophagogastroduodenoscop* OR "capsule endoscop*") AND TITLE-ABS-KEY (image* OR video* OR "real time" OR automated) AND TITLE-ABS-KEY (esophag* OR gastric OR stomach OR duoden* OR Barrett* OR varice* OR ulcer* OR "early gastric cancer") AND PUBYEAR < 2026
Embase
('artificial intelligence':ti,ab,kw OR AI:ti,ab,kw OR 'machine learning':ti,ab,kw OR 'deep learning':ti,ab,kw OR 'neural network*':ti,ab,kw OR 'convolutional neural network*':ti,ab,kw OR CNN:ti,ab,kw OR 'computer aided detection':ti,ab,kw OR 'computer aided diagnosis':ti,ab,kw OR CADe:ti,ab,kw OR CADx:ti,ab,kw OR 'clinical decision support':ti,ab,kw) AND (endoscop*:ti,ab,kw OR 'upper endoscopy':ti,ab,kw OR gastroscop*:ti,ab,kw OR esophagogastroduodenoscop*:ti,ab,kw OR EGD:ti,ab,kw OR 'capsule endoscop*':ti,ab,kw OR 'video capsule':ti,ab,kw OR VCE:ti,ab,kw OR 'endoscopic ultrasound':ti,ab,kw OR EUS:ti,ab,kw) AND (esophag*:ti,ab,kw OR 'esophageal cancer':ti,ab,kw OR 'esophageal neoplasm*':ti,ab,kw OR 'esophageal squamous cell carcinoma':ti,ab,kw OR ESCC:ti,ab,kw OR adenocarcinoma:ti,ab,kw OR Barrett*:ti,ab,kw OR 'early esophageal cancer':ti,ab,kw) AND (image*:ti,ab,kw OR video*:ti,ab,kw OR 'real time':ti,ab,kw OR automated:ti,ab,kw) AND [1000-2026]/py

Supplementary File S3. Main methodological limitations of included studies

Author	Main limitations
Wang J et al. [22]	Limited multicenter magnification datasets; incomplete IPCL subtype detection
Meng QQ et al. [23]	Static images only; limited endoscopist representation
Zhao Z et al. [24]	Single-center design; dependence on NBI datasets
Feng Y et al. [25]	Static images; no invasion depth assessment
Wang YK et al. [26]	Small sample size; standard-resolution endoscopy
Shiroma S et al. [27]	Limited video conditions; high-resolution equipment only
Uema R et al. [28]	Small and imbalanced dataset; no prospective validation
Wang YK et al. [29]	Small sample; high cost of hyperspectral imaging
Chou CK et al. [30]	Limited sample size; static images
Horie Y et al. [31]	Small adenocarcinoma subgroup; retrospective design
Tang D et al. [32]	ESCC only; no invasion-depth analysis
Guo L et al. [33]	No prospective clinical validation
Yuan XL et al. [34]	Potential selection bias in edited images/videos
Yuan XL et al. [35]	Limited evidence on cost-effectiveness
Li SW et al. [36]	Population ≥50 years; lack of gold standard
Ohmori M et al. [37]	Selected high-quality images only
Aoyama N et al. [38]	Single-center design; no real-time validation
Tajiri A et al. [39]	Excluded Barrett-associated adenocarcinoma
Li B et al. [40]	Single-center study; no fatigue control
Tani Y et al. [41]	No invasion-depth analysis
Gao X et al. [42]	No real-time video validation
Yang XX et al. [43]	No clinical-practice validation
Yuan XL et al. [44]	Imbalanced IPCL subtype distribution
Tang S et al. [45]	Single-hospital dataset
Waki K et al. [46]	High ESCC prevalence in validation videos
Li B et al. [47]	Limited image scale; selected datasets
Everson MA et al. [48]	Further real-time validation required
Tang et al. [49]	Limited lesion diversity
Cai et al. [50]	Other lesion types not evaluated
Yuan XL et al. [51]	Limited video dataset

Notes: ESCC, esophageal squamous cell carcinoma; IPCL, intrapapillary capillary loops; NBI, narrow-band imaging; WLE, white light endoscopy; ME-NBI, magnifying endoscopy with narrow-band imaging; BLI, blue light imaging; HSI, hyperspectral imaging.
